# Accuracy of transabdominal ultrasound to diagnose functional constipation and fecal impaction in children: a systematic review and meta-analysis

**DOI:** 10.1007/s00247-024-06083-4

**Published:** 2024-11-15

**Authors:** Johanna M. B. W. Vos, Michelle N. Bloem, Anna de Geus, Mariska M. G. Leeflang, René Spijker, Ilan J. N. Koppen, Desiree F. Baaleman, Marc A. Benninga

**Affiliations:** 1 Department of Pediatric Gastroenterology and Nutrition, Emma Children’s Hospital, Amsterdam UMC, University of Amsterdam, Meibergdreef 9, 1105AZ, Amsterdam, Netherlands; 2 Amsterdam Gastroenterology Endocrinology Metabolism Research Institute, Amsterdam UMC, Amsterdam, Netherlands; 3 Department of Epidemiology and Data Science, Amsterdam UMC, Amsterdam Public Health, University of Amsterdam, Amsterdam, Netherlands; 4 Amsterdam Public Health, Medical Library, Amsterdam UMC, University of Amsterdam, Amsterdam, Netherlands; 5 Amsterdam Reproduction and Development Research Institute, Amsterdam UMC, Amsterdam, Netherlands

**Keywords:** Constipation, Fecal impaction, Pediatrics, Rectum, Ultrasonography

## Abstract

**Background:**

Functional constipation is common in children and accurate diagnostic methods are essential for early diagnosis and effective management. The diagnostic accuracy of transabdominal ultrasound to diagnose functional constipation is unclear.

**Objective:**

To evaluate the diagnostic accuracy of transverse rectal diameter measurement via transabdominal ultrasound in diagnosing children with functional constipation and in identifying fecal impaction.

**Materials and methods:**

Electronic databases were searched from inception to March 2023. Original studies investigating the diagnostic accuracy of measuring transverse rectal diameter via transabdominal ultrasound, including children with and without functional constipation, or with and without fecal impaction were included. Data extraction and quality assessment were performed independently by two reviewers.

**Results:**

Sixteen studies were included (*n* = 1,801 children, 0–17 years). Thirteen studies investigated the diagnostic accuracy for functional constipation, and five for fecal impaction. High risk of bias was found across the majority of studies mainly due to un-blinded case–control designs. Cut-off transverse rectal diameter values to diagnose functional constipation ranged from 2.4 cm to 3.8 cm. Meta-analysis (seven studies, *n* = 509 children) estimated mean sensitivity and specificity to diagnose functional constipation were 0.68 (95% confidence interval (CI) 0.55–0.78) and 0.81 (95% CI 0.71–0.88), respectively. Meta-analysis of diagnostic accuracy of identifying fecal impaction was not feasible. Studies reported a sensitivity and specificity ranging between 68–100% and 83–100%, respectively.

**Conclusion:**

Transabdominal ultrasound may be a valuable non-invasive diagnostic tool to diagnose functional constipation by measuring transverse rectal diameter and identifying fecal impaction in children. Heterogeneous study methods and lack of age-dependent normal values impair current clinical recommendations. Future research should focus on separating age groups and developing a standardized protocol.

**Graphical Abstract:**

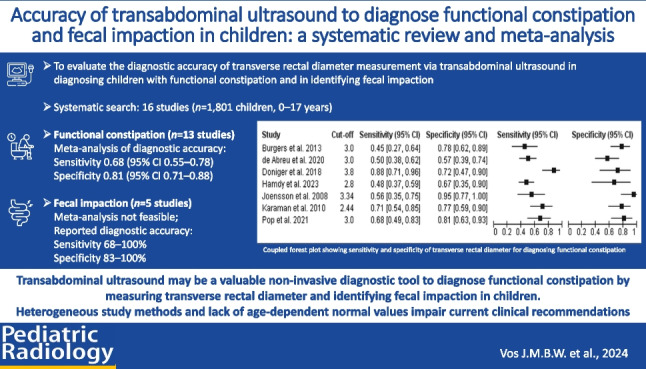

**Supplementary Information:**

The online version contains supplementary material available at 10.1007/s00247-024-06083-4.

## Introduction

Functional constipation is a common problem in children and adolescents, with a worldwide pooled prevalence of 9.5% and is characterized by hard, painful, and infrequent bowel movements without an underlying organic cause (e.g., anatomic, endocrine, neurologic, or other diseases) [1]. To diagnose a child with functional constipation, the child must fulfill at least two of six Rome IV criteria (Table 1) [2, 3]. One of these criteria is the presence of a large rectal fecal mass, also referred to as fecal impaction. International guidelines recommend treatment of fecal disimpaction using a short-term high dose of laxatives, followed by maintenance therapy with laxatives [4].

**Table 1:** Rome IV criteria for functional constipation

I. **Rome IV criteria for functional constipation in infants and toddlers up to 4 years old** [2]
Must include two or more of the following present for at least one month: 1. Two or fewer defecations per week 2. History of excessive stool retention 3. History of painful or hard bowel movements 4. Presence of a large diameter stools 5. History of large fecal mass in the rectum In toilet-trained children, the following additional criteria may be used: 6. At least one episode/week of incontinence after the acquisition of toileting skills 7. History of large-diameter stools that may obstruct the toilet
II. **Rome IV criteria for functional constipation in children & adolescents (developmental age ≥ 4 years)** [2]
Must include two or more of the following occurring at least once per week for a minimum of one month with insufficient criteria for a diagnosis of irritable bowel syndrome^a^: 1. Two or fewer defecations in the toilet per week 2. At least one episode of fecal incontinence per week 3. History of retentive posturing or excessive volitional stool retention 4. History of painful or hard bowel movements 5. Presence of a large fecal mass in the rectum 6. History of large diameter stools that can obstruct the toilet

After appropriate evaluation, the symptoms cannot be fully explained by another medical condition

^a^*Rome IV criteria*^b^ *for irritable bowel syndrome (IBS) in children and adolescents must include all of the following:*

1. Abdominal pain at least four days per month associated with one or more of the following:

a. Related to defecation

b. A change in frequency of stool

c. A change in form (appearance) of stool

2. In children with constipation, the pain does not resolve with resolution of the constipation (children in whom the pain resolves have functional constipation, not irritable bowel syndrome)

3. After appropriate evaluation, the symptoms cannot be fully explained by another medical condition

^b^Criteria fulfilled for at least two months before diagnosis

Digital rectal examination may be used to identify a large rectal fecal mass to evaluate the need for disimpaction in children with functional constipation [5, 6]. International guidelines recommend to perform digital rectal examination if the diagnosis of functional constipation is uncertain, if only one of the diagnostic criteria is present, or in the presence of alarm signs or symptoms (e.g., ribbon stools) to exclude underlying medical conditions [4]. Measurement of the rectal filling state with digital rectal examination is controversial; physicians find the test unpleasant, too invasive, and not child-friendly. Digital rectal examination is contra-indicated in children refusing examination or presenting with negative psychological factors, such as severe anxiety or a history of sexual abuse [7]. Therefore, a non-invasive diagnostic modality can be of additional value in diagnosing functional constipation or fecal impaction. A previous systematic review (2012) concluded that there was insufficient evidence for using abdominal radiography, colonic transit time, and ultrasound to diagnose pediatric functional constipation [8]. Since then, international guideline explicitly states against using abdominal radiography to diagnose functional constipation [4]. A recent consensus paper suggests that colonic transit time may be used in the further characterization of constipation, but not in its initial diagnosis [9]. Besides poor diagnostic accuracy, both abdominal radiography and colonic transit time cause radiation exposure. To date, transabdominal ultrasound is widely used in general pediatrics and several studies have been conducted in which transabdominal ultrasound is proposed as a diagnostic tool for functional constipation. Children with functional constipation may have an increased rectal diameter as a result of long-term fecal accumulation, and transverse rectal diameter measurement via transabdominal ultrasound could help distinguish children with functional constipation from healthy children [8]. Transabdominal ultrasound is a point of care tool, safe, and reproducible, and therefore allows for monitoring treatment responses over time.

In order to provide recommendations on the role of transabdominal ultrasound in the diagnostic pathway, a comprehensive overview of the existing literature is necessary. This study provides an updated systematic review of current literature regarding the use of transabdominal ultrasound in children with functional constipation. Our primary objective was to assess the diagnostic accuracy of transverse rectal diameter measurement via transabdominal ultrasound (index test) compared with the Rome/IOWA criteria for functional constipation (reference test). Secondary objectives were to assess the diagnostic accuracy of transverse rectal diameter via transabdominal ultrasound (index test) compared with the digital rectal examination (reference test) to assess fecal impaction and to compare mean transverse rectal diameter between children with and without functional constipation.

## Methods

This systematic review was registered at the International Prospective Register of Systematic Reviews (PROSPERO) with registration number CRD42022355421 and is reported in accordance with the PRISMA statement [10].

### Search strategy and study selection

The EMBASE and MEDLINE databases were searched from inception to March 2023. Search terms included, but were not limited to the following: “constipation,” “Rome,” “ultrasound,” and synonyms (the full search strategy is provided in Supplementary Material 1). Studies were eligible if they met the following criteria: (1) original studies of diagnostic accuracy (observational studies, trials) published in a peer-reviewed journal in English or Dutch; (2) the study evaluated the diagnostic accuracy of transverse rectal diameter measurement via transabdominal ultrasound compared to diagnosing a child with functional constipation, or assessing fecal impaction via digital rectal examination; (3) the study population consisted of children with and without functional constipation and/or fecal impaction from 0 to 17 years of age, including all age ranges; (4) functional constipation was defined according to the pediatric Rome II, III, or IV or IOWA criteria [2, 3, 11–13]. Studies were excluded if they included children with an organic cause of constipation or a surgical history of the gastro-intestinal tract. Two reviewers (A.G. and J.V.) independently screened all titles and abstracts for selection with the use of Rayyan [14]. Full-text review and data extraction were executed independently by two authors (A.G. and J.V.). Any disagreements were resolved upon mutual agreement. In case of persistent disagreement, a third reviewer was consulted (D.B.).

### Data extraction

Extracted data included general study details (study design; study setting); population information (inclusion and exclusion criteria, sample size, age, sex distribution, definition of cases and controls, possible treatment for functional constipation); method used to measure transverse rectal diameter via transabdominal ultrasound (frequency, placement, and angle of the transducer, number of measurements, bladder filling state, and time to last defecation [15]); method used to diagnose functional constipation and/or perform digital rectal examination; blinding of outcome assessors; outcomes of diagnostic test accuracy testing (sensitivity, specificity, true positive (TP), false positive (FP), false negative (FN), and true negative (TN) counts); and mean/median values of transverse rectal diameters in children with and without functional constipation. If studies did not directly report all data for TP, FP, FN, and TN, the remaining values were calculated based on the reported data when feasible; otherwise, authors were contacted to provide additional data. No assumptions were made.

### Quality assessment

Study quality was assessed by two authors (J.V. and D.B.) using the Quality Assessment of Diagnostic Accuracy Studies-2 (QUADAS-2) [16]. The QUADAS-2 tool assesses the risk of bias and concerns regarding applicability (high, low, or unclear). It examines four bias domains: patient selection, index test, reference standard, and flow/timing. Risk of bias was scored as low if “yes” was answered for all signaling questions, high if “no” was reported for at least one signaling question, and unclear if “unclear” was scored for any signaling question. Within the first three domains, study applicability for each study was assessed. Any disagreements in the assessment were resolved by a third author. A detailed description is available in Supplementary Material 2. Quality assessment was performed for both functional constipation and fecal impaction separately if both were reported in the same study.

### Statistical analysis

A coupled forest plot was developed with 95% CI for sensitivity and specificity among studies with functional constipation as the index test. A bivariate meta-analysis for studies with a transverse rectal diameter cut-off at 3.0 cm was conducted to provide summary estimates of sensitivity and specificity at that threshold. Because the reported cut-off of an abnormal transverse rectal diameter for an functional constipation diagnosis differed among the studies, an adjusted transverse rectal diameter cut-off was calculated relatively to 3.0 cm and examined as a covariate in the meta-analysis. If sufficient data were available, we planned to conduct bivariate meta-analyses only including studies which had excluded children who had defecated within 2 h prior to the examination [15].

Separate analyses were conducted for functional constipation (according to Rome IV criteria) and fecal impaction (defined after digital rectal examination). In case of insufficient data for meta-analyses, studies were analyzed in a descriptive manner.

Difference in mean transverse rectal diameter among children with and without functional constipation was examined by comparing pooled mean transverse rectal diameters and by calculating a standardized mean difference (SMD) with a 95% CI [17]. If medians were provided, the mean and standard deviation were estimated from the median, range, and sample size using the formula as proposed by Hozo et al. [18].

If risk of bias analysis allowed it (see QUADAS-2), we aimed to perform a sensitivity analysis based on studies with low risk of bias.

Analyses were conducted and made in R using the packages “meta,” “mada,” “metafor,” “robvis,” “lme,” and “dmetar,” and a hands-on guide [19–23]. Review Manager 5.4 (Review Manager (RevMan) Version 5.4, The Cochrane Collaboration, 2020, London, UK) was used to generate forest plots for primary and secondary outcomes with 95% CIs.

## Results

**Fig. 1:**
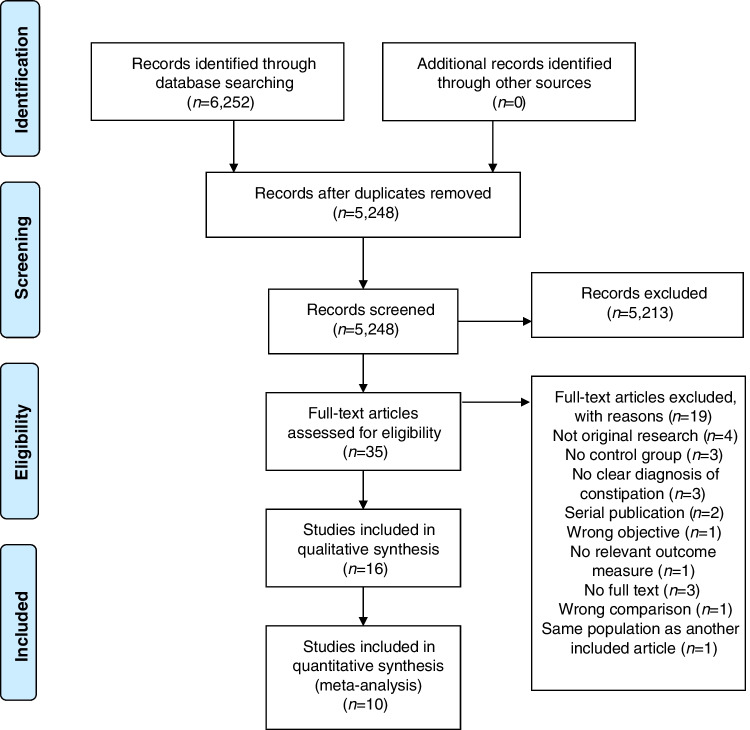
PRISMA flowchart

A total of 6,252 studies were identified, of which 16 were eligible for inclusion. The PRISMA flowchart is depicted in Fig. 1 and shows the reasons for exclusion. Eleven were case–control studies [15, 24–33] and five were prospective case–control studies [34–38]; see Table 2. The control groups consisted of healthy controls with normal defecation patterns and of children with gastro-urinary symptoms not fulfilling criteria for functional constipation (see Table 2). Altogether, these studies included 1,801 children aged between 0 and 17 years old. Thirteen of sixteen studies reported sex of whom 686/1,524 (45%) children were female [15, 24–27, 29, 31, 32, 34–38]. The sample sizes of the studies ranged from 28 to 304 children. Nine studies (56%) were conducted in tertiary care [24–26, 28, 31–34], six (38%) in secondary care [15, 29, 35–38], and one (6%) was conducted in both tertiary and secondary care [27]. Studies were conducted in Europe (*n* = 8) [15, 24, 28, 30, 32–34, 38], Asia (*n* = 4) [25, 27, 29, 31], North America (*n* = 1) [36], South America (*n* = 1) [35], and North Africa (*n* = 1; Table 2) [26].

**Table 2:** Study characteristics

Study (author, year, country)	Design	Setting	Reference test	Cases	Control	Blinding	*n*	Age in years	Female, *n* (%)
Klijn 2004, The Netherlands [30]	Case–control	3rd, pediatric urology	IOWA criteria	FC diagnosis based on IOWA criteria and voiding dysfunction	Urological patients with normal defecation pattern and no LUTD	Unclear	49	Range all children, 5–13	NR
Singh 2005, UK [33]	Case–control	3rd, pediatric surgery	IOWA criteria	FC diagnosis	No history of bowel problems	Unclear	177	Median patients, 6.5 (IQR 0.4–16.4) Median controls, 5.5 (IQR 0.3–15.3)	NR
Bijos 2007, Poland [24]	Case–control	3rd, pediatric gastroenterology	Rome II	FC diagnosis	Normal defecation pattern	Unclear	225	Mean all children, 6.3 (range, 1.6–17.8)	48 (40%)
Joensson 2008, Denmark [28]	Case–control	3rd, pediatric department	Rome III	FC diagnosis	RC-III < 2	No	51	Mean patients, 7.2 (SD 1.8) Mean controls, 9.1 (SD 2.7)	NR
			DRE	With and without FC, with FI	With and without FC, without FI	Unclear	48	NR	NR
Karaman 2010, Turkey [29]	Case–control	2nd, pediatric surgery	Rome III	FC diagnosis	RC-III < 2	Yes	66	Mean patients, 6.8 (SD 2.9) Mean controls, 8.4 (SD 3.8)	27 (41%)
Burgers 2013, The Netherlands [34]	Cross-sectional	3rd, pediatric urological surgery	Rome III	FC diagnosis (specific urinary symptoms not reported)	RC-III < 2 (specific urinary symptoms not reported)	Unclear	84	Median all children, 9.0 (IQR 6.4–11.0)	30 (36%)
			DRE	With and without FC, with full rectum (specific urinary symptoms not reported)	With and without FC, with empty or half-filled rectum (specific urinary symptoms not reported)	Yes	84	Median all children, 9.0 (IQR 6.4–11.0)	30 (36%)
Modin-Dalby 2015, Denmark [15]	Case–control	2nd, pediatric department	Rome III	FC diagnosis	No history of FC, UTI, or laxative use	No	28	Median patients, 7.0 (IQR 6.0–8.5) Median control 7.0 (5.5–10.5)	11 (39%)
Modin-Walsted 2015, Denmark [38]	Cross-sectional	2nd, pediatric department	DRE	With FC based on Rome III and with FI	With FC, but without FI	No	225	Median all children, 6.4 (IQR 2.0–15.8)	113 (48%)
Hatori 2017, Japan [27]	Case–control	2nd and 3rd, pediatric gastroenterology	DRE	With and without FC based on Rome III with FI	With and without FC (Rome III), but without FI	Unclear	100	Median all children, 5.0 (IQR 0.0–15.0)	47 (47%)
Doniger 2018, USA [36]	Cross-sectional	2nd, pediatric emergency department	Rome III	FC diagnosis	With clinical suspicion of FC, but RC-III < 2	Yes	50	Mean patients, 9.5 (SD 3.5) Mean controls, 10.5 (SD 3.9)	32 (64%)
Momeni 2019, Iran [31]	Case–control	3rd, pediatric gastroenterology	Rome III	FC diagnosis	RC-III < 2	No	76	Unclear	32 (42%)
De Abreu 2020, Brazil [35]	Cross-sectional	2nd, pediatric urology	Rome IV	FC diagnosis with LUTS	RC-IV < 2 with LUTS	No	107	Mean all children, 8.9 (SD 3.2)	59 (55%)
Pop 2021, Romania [32]	Case–control	2nd and 3rd, pediatric department	Rome III/IV	FC diagnosis based on Rome IV	RC-III-IV < 2	Unclear	65	Mean patients, 5.4 (SD 4.3) Mean controls, 5.6 (SD 3.6)	29 (45%)
Imanzadeh 2022, Iran [37]	Cross-sectional	2nd, pediatric gastroenterology	DRE	Rome IV and FI	Rome IV without FI	No	94	Mean all children, 5.5 (SD 3.2)	41 (44%)
Dogan 2022, Turkey [25]	Case–control	3rd, pediatric gastroenterology	FC diagnosis	Rome IV	RC-IV < 2	Unclear	304	Mean patients, 8.1 (SD 5.2) Mean controls, 8.5 (SD 5.2)	172 (57%)
Hamdy 2023, Egypt [26]	Case–control	3rd, pediatric gastroenterology	FC diagnosis	Rome IV	Not suffering from any constipation symptoms	Unclear	100	Median patients, 7 (IQR 5–7) Median controls, 6 (IQR 4–8)	44 (44%)

*DRE* digital rectal examination, *FC* functional constipation, *FI* fecal impaction, *IQR* interquartile range, *LUTD* lower urinary tract dysfunction, *LUTS* lower urinary tract symptoms, *NR* not reported, *RC* Rome criteria, *SD* standard deviation

### Rectal diameter measurement methods

Methods to measure transverse rectal diameter via transabdominal ultrasound varied among studies (Table 3). Ten studies (63%) used the method described by Klijn et al. which includes measurements of the transverse rectal diameter with the ultrasound probe 2 cm above the symphysis at an angle of 15° [30]. Bladder filling during assessment varied between empty (*n* = 1) [29], partially filled (*n* = 9) [24–26, 28, 30, 33–35, 37], and full bladder (*n* = 2) [27, 31]. In four studies, these details were not adequately described [15, 32, 36, 38]. Usually the measurement was performed two to three times, and the mean transverse rectal diameter was considered the correct transverse rectal diameter. Time to defecation prior to the transabdominal ultrasound was reported in nine (56%) studies [15, 24, 26, 28–30, 34, 35, 38]. In two of these studies, children were excluded if they defecated within 12 h prior to the ultrasound [24, 29]. In three studies, the ultrasound was postponed if children felt the urge to defecate [15, 35, 38]. In four studies, both conditions (urge to defecate and time to previous defecation) were considered [26, 28, 30, 34]. The cut-off for an enlarged transverse rectal diameter varied between 2.44 cm and 3.80 cm across studies. Four studies used a pre-defined cut-off value of 3 cm [15, 34, 35, 38]. Four studies determined the optimal cut-off by modeling the area under the ROC curve based on their collected data [26, 27, 29, 36]. Two studies determined the cut-off by either using the mean of the controls + 2 SD or by the 75th percentile of the controls [28, 33]. None of the studies reported sensitivity and specificity at multiple thresholds.

**Table 3:** Transverse rectal diameter assessment and outcomes for diagnosing functional constipation

Study	Placement	Angle (°)	Bladder filling state	Time since last defecation	Number of measurements	Threshold TRD in cm	Sensitivity (95% CI)	Specificity (95% CI)	TRD cases in cm, mean (SD) or median (IQR)	TRD controls in cm, mean (SD) or median (IQR)
Klijn 2004 [30]	2 cm above symphysis	15	30–70% filled	> 2 h	2	NR	-	-	4.9 (1.0)	2.1 (0.6)
Singh 2005 [33]	1–2 cm above symphysis	90	Partially filled	NR	NR	3.00^d^	-	-	3.4 (2.1–7.0)^f^	2.4 (1.3–4.2)^f^
Bijos 2007 [24]	NR	NR	Slightly filled	> 12 h	Several times	NR	-	-	4.3 (1.0)	3.2 (0.8)
Joensson 2008 [28]	2 cm above symphysis	10–15	Partially filled	> 3 h	3	3.34^c^	56	96	4.0 (0.8)	2.1 (0.6)
Karaman 2010 [29]	NR	NR	Empty	> 12 h	2	2.44^b,e^	71	76	3.4 (1.0)	2.1 (0.7)
Burgers 2013 [34]	2 cm above symphysis	15–20	30–70% filled	NR	2	3.00^a^	45	78	2.8 (2.1–3.8)^f^	2.4 (2.0–2.9)^f^
Modin-Dalby 2015 [15]	2 cm above symphysis	15	NR	> 0 h	3	3.00^a^	-	-	3.6 (0.6)	1.9 (0.5)
Doniger 2018 [36]	2 cm above symphysis	15	Not specified	NR	2	3.80^b^	86	71	4.3 (1.4)	2.9 (1.2)
Momeni 2019 [31]	NR	NR	Filled	NR	3	NR	-	-	3.2 (1.0)	2.0 (0.4)
De Abreu 2021 [35]	2 cm above symphysis	15	30–70%	NR	NR	3.00^a^	50	57	3.0 (0.8)	2.9 (1.0)
Pop 2021 [32]	Pelvis level posterior to the bladder	NR	No criterion	NR	NR	3.00 (unclear if pre-specified or not)	68	81	3.6 (1.4)	2.4 (0.7)
Dogan 2022 [25]	Above symphysis	NR	Partially filled	NR	NR	NR	^g^	^g^	^g^	^g^
Hamdy 2023 [26]	Anterior abdomen above symphysis	15	Partially filled	> Few hours	3	2.80^b^	84	92	3.6 (3.2–4.0)^f^	2.3 (1.8–2.5)^f^

*CI* confidence interval, *IQR* interquartile range, *NR* not reported, *TRD* transverse rectal diameter, *SD* standard deviation

^a^Pre-specified

^b^Not pre-specified, based on area under the curve

^c^Not pre-specified, based on mean control group + 2 SD

^d^Not pre-specified, based on 75th percentile control group

^e^Post-miction

^f^Median

^g^Dogan et al. reported data separated into four different age groups and diagnosis (functional constipation vs control), and presence or absence of fecal mass. A meta-analysis within this study was performed and used in the meta-analysis of this systematic review

### Quality assessment

**Fig. 2:**
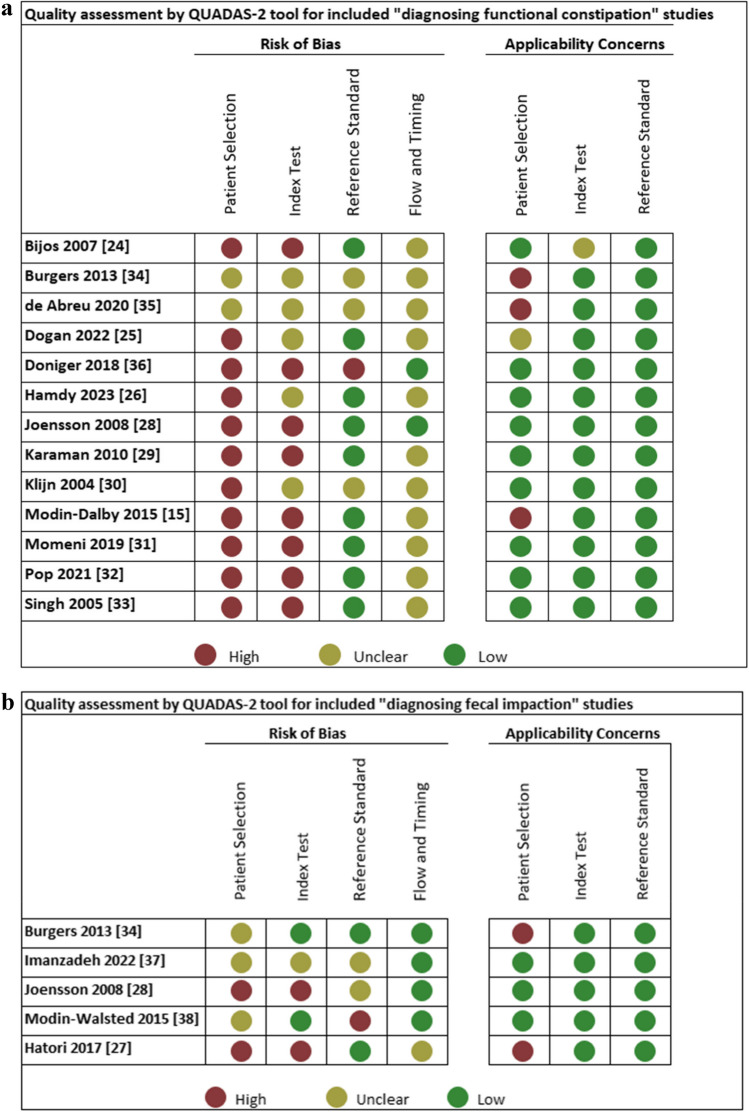

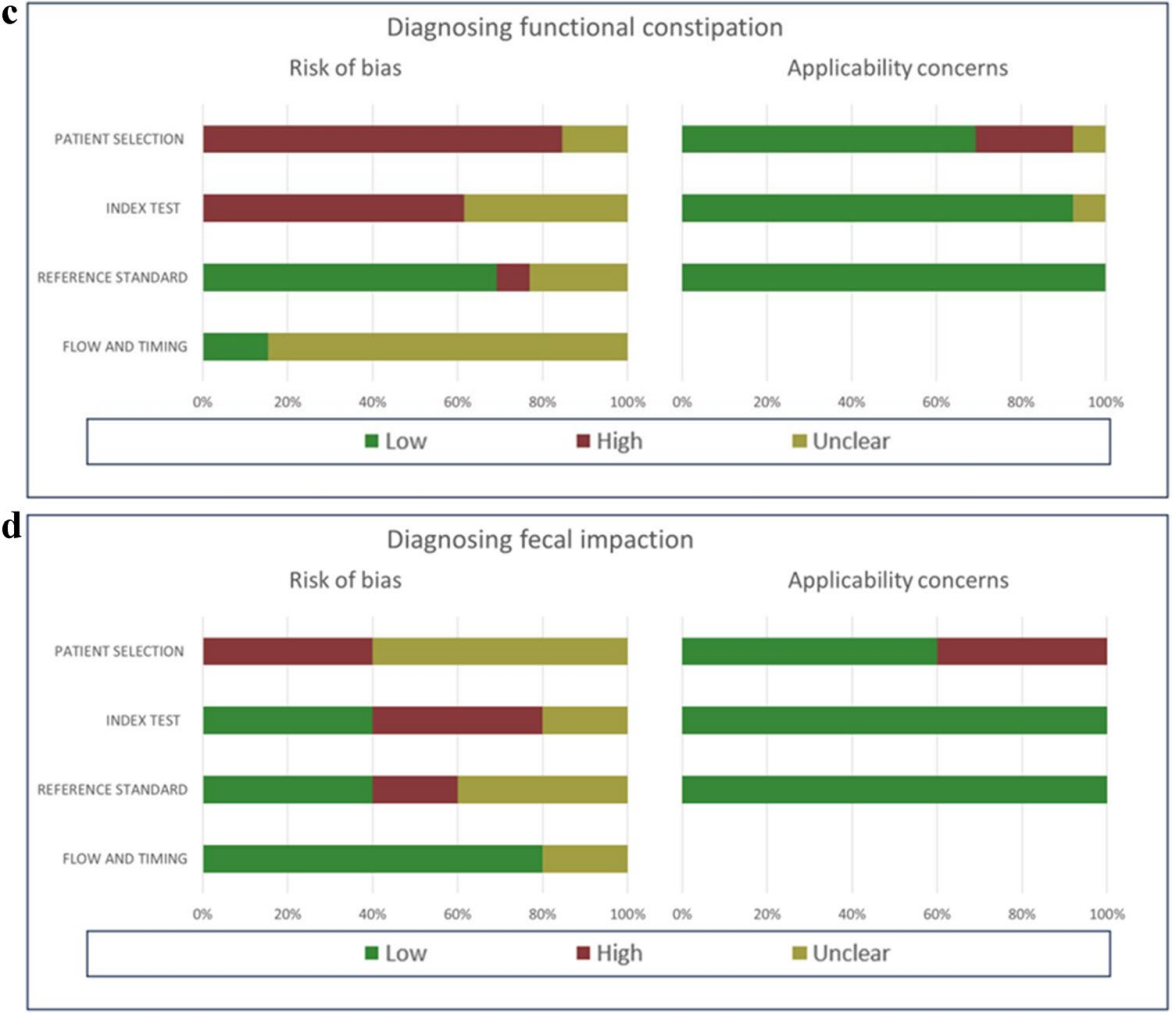
**a** Quality assessment of included studies for diagnosing functional constipation. **b** Quality assessment of included studies for diagnosing fecal impaction. **c** Summary of quality assessment of included studies for diagnosing functional constipation. **d** Summary of quality assessment of included studies for diagnosing fecal impaction

QUADAS-2 assessments of the included studies are depicted in Fig. 2. All but four studies were assessed as having a high risk of bias for patient selection, mainly caused by the case–control study designs. Four studies were judged as unclear, due to uncertainty about consecutive patient enrollment [34, 35, 37, 38]. Concerning the measurement of the index test, six studies were judged as having an unclear risk of bias [25, 26, 30, 34, 35, 37]. This was because most studies were performed un-blinded and outcomes of the reference test (Rome criteria) were known before performance of the index test (ultrasound). Applicability concerns were raised in three studies, in which the index group used polyethylene glycol, and the control group included patients treated for functional constipation or patients with urological symptoms [15, 34, 35].

### Diagnostic accuracy of transverse rectal diameter measurement to diagnose functional constipation

**Fig. 3:**
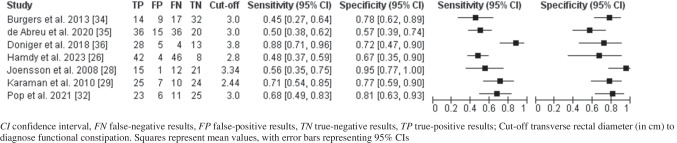
Coupled forest plot showing sensitivity and specificity of transverse rectal diameter for diagnosing functional constipation

Thirteen studies assessed the diagnostic accuracy of transverse rectal diameter measurement via transabdominal ultrasound (index test) compared to functional constipation diagnosis (reference test) [15, 24–26, 28–36]. Functional constipation was diagnosed according to the Rome II criteria (*n* = 1; 8%) [24], the Rome III criteria (*n* = 6; 46%) [15, 28, 29, 31, 34, 36], the Rome IV criteria (*n* = 3; 23%) [25, 26, 35], Rome III and Rome IV criteria (*n* = 1; 8%) [32], or the IOWA criteria (*n* = 2; 15%) [30, 33]. We included seven studies in the meta-analysis, including 281 children with functional constipation and 228 children without functional constipation [26, 28, 29, 32, 34–36]. Figure 3 depicts the coupled forest plot. Using logistic regression analysis, the sensitivity was determined at 0.68 (95% CI 0.55–0.78) and specificity at 0.81 (95% CI 0.71–0.88).

Studies used different cut-off values to diagnose functional constipation; these varied between 2.44 cm and 3.8 cm (Table 2). Because the reported cut-off of an abnormal transverse rectal diameter for a functional constipation diagnosis differed among the studies, an adjusted transverse rectal diameter cut-off was calculated relatively to 3.0 cm and examined as a covariate in the meta-analysis. Logistic regression meta-analysis of included studies using the adjusted transverse rectal diameter thresholds found similar estimates of sensitivity (0.67; 95% CI 0.55–0.78) and specificity (0.81; 95% CI 0.71–0.88). Based on this meta-analysis, a lower or higher transverse rectal diameter cut-off did not have a statistically significant association with the sensitivity or specificity of the model.

### Diagnostic accuracy of transverse rectal diameter measurement to diagnose fecal impaction

Five studies assessed the diagnostic accuracy of transverse rectal diameter measurement via transabdominal ultrasound (index test) compared to fecal impaction determination via digital rectal examination (reference test) [27, 28, 34, 37, 38]. These studies included 332 children with fecal impaction and 219 children without fecal impaction. Three studies included both children with functional constipation and without functional constipation in their population [27, 28, 34]. Two studies included only children with functional constipation (*n* = 94) [37, 38]. It was not feasible to conduct a meta-analysis due to the low number of studies, each with variance in reporting their results and heterogeneous study populations. Transverse rectal diameter thresholds ranged between 2.7 cm and 3.0 cm. Reported sensitivity and specificity to diagnose fecal impaction via transverse rectal diameter measurement ranged between 68–100% and 83–100%, respectively. Three studies reported a significant higher mean transverse rectal diameter in children with fecal impaction compared to children without fecal impaction. The other two studies did not perform statistical analyses to compare means between groups. Additional study characteristics and outcomes are described in Table 4.

**Table 4:** Transverse rectal diameter assessment and outcomes for diagnosing fecal impaction

Study	Placement	Angle (°)	Bladder filling state	Time since last defecation	Number of measurements	Threshold TRD in cm	TP	FP	FN	TN	Sensitivity (95% CI)	Specificity (95% CI)	TRD cases in cm, mean (SD) or median (IQR)		TRD controls in cm, mean (SD) or median (IQR)
Joensson 2008 [28]	2 cm above symphysis	10–15	Partially filled	> 3 h	3	2.94^c^	22	0	0	26	100	100	4.1 (0.8)	2.1 (0.4)	
Burgers 2013 [34]	2 cm above symphysis	15–20	Moderately filled	NR	2	3.00^a^	23	4	11	46	68	92	3.3 (2.8–3.9)^d^	Half-filled rectum, 2.5 (1.8–2.8)^d^ Empty rectum, 2.0 (1.5–2.2)^d^	
Modin-Walsted 2015 [38]	2 cm above symphysis	15	No criterion	> 0 h	NR	3.00^a^	132	13	17	63	89	83	3.4 (0.6)	2.2 (0.6)	
Hatori 2017 [27]	2 cm above symphysis	15	Filled bladder	NR	3	2.70^b^	63	2	3	32	96	94	-	-	
Imanzadeh 2022 [37]	2 cm above symphysis	NR	Half filled	NR	NR	NR	-	-	-	-	-	-	3.9 (1.0	2.9 (1.2)	

*CI* confidence interval, *FN* false negative, *FP* false positive, *IQR* interquartile range, *NR* not reported, *TN* true negative, *TP* true positive, *TRD* transverse rectal diameter, *SD* standard deviation

^a^Pre-specified

^b^Not pre-specified, based on area under the curve

^c^Not pre-specified, based on mean control group + 2 SD

^d^Median

### Differences in mean transverse rectal diameter between children with and without functional constipation

**Fig. 4:**
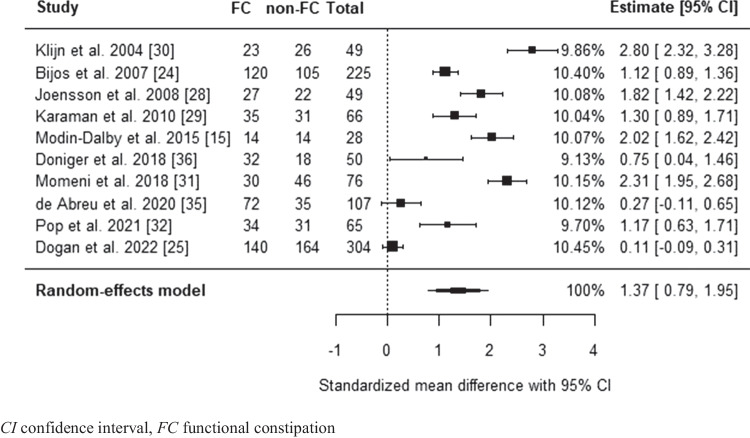
Forest plot of standardized mean difference in transverse rectal diameter between children with functional constipation and without functional constipation

The mean transverse rectal diameter of children with and without functional constipation was reported in all studies (*n* = 13) which assessed the diagnostic accuracy of transverse rectal diameter measurement via transabdominal ultrasound compared to functional constipation diagnosis [15, 24–26, 28–36]. For one study, a meta-analysis within the study was performed on the mean transverse rectal diameter with and without functional constipation across different age groups in the presence and absence of fecal mass prior to comparing pooled mean transverse rectal diameter across all studies [25]. Random-effects meta-analysis estimated the overall effect size (SMD) to be 1.37, with a standard error (SE) of 0.30 (95% CI 0.79–1.95; *P* < 0.0001; Fig. 4). Mean transverse rectal diameter for the functional constipation group was estimated at 3.77 cm ± 0.29 SD, versus 2.21 cm ± 0.21 SD for the non-functional constipation group. A meta-analysis on differences in transverse rectal diameter was also performed including three studies reporting median transverse rectal diameter [26, 33, 34], after conversion of median-to-mean following Hozo et al. [18]. Upon addition of the three studies, the estimated overall effect size (SMD) remained significant and within the same range as the model based on the studies reporting a mean transverse rectal diameter.

### Factors impacting transverse rectal diameter measurements

The included studies described several factors which could have influenced the transverse rectal diameter measurements.

### Age and sex

Age was discussed in 11 studies [15, 24–29, 32–34, 36]. Among these studies, six found a positive correlation between age and transverse rectal diameter [24–26, 29, 32, 33], while five showed no correlation [15, 27, 28, 34, 36]. Only two of these studies provided mean transverse rectal diameter per four age categories; under 3 years, 3–6 years, 6–12 years, and over 12 years old [24, 25]. In general, the studies which found a correlation between age and transverse rectal diameter had a larger sample size with a wider age range [24, 25, 33]. The correlation between transverse rectal diameter and sex was explored in four studies, but no significant associations were identified [26, 28, 29, 31].

### Technique to measure transverse rectal diameter

Twelve studies performed transverse rectal diameter measurements 2 cm above the symphysis pubis with an angle of 10–15° as described by Klijn et al. [15, 25–28, 30, 33–38]. Two studies performed transverse rectal diameter measurements in three different planes (above symphysis pubis, under ischial spine, and bladder neck) [25, 27]. One study described that the highest proportion of measurable transverse rectal diameter in subjects was at the transection located above the symphysis [27]. Another study reported significant differences between constipated children and controls in all planes [25].

### Intra- and inter-observer variabilities

Intra- and inter-observer variabilities were reported in three studies [28, 29, 34]. Two studies showed a small intra-observer variability, with a coefficient of variation of 5.8% and an absolute intraclass correlation coefficient of 0.94, respectively [28, 34]. Others did not show any inter-observer differences between ultrasound measurements of two radiologists [29].

### Time of defecation and effect of treatment

Time to prior defecation was inadequately reported in most studies. Only four studies excluded patients based on time to prior defecation, varying from 2 to 24 h [24, 28–30]. The use of laxatives was inadequately reported in most studies. All studies evaluating treatment response showed transverse rectal diameter decreased after treatment [15, 26, 28, 36, 37]. However, this trend was not statistically significant in two studies [29, 36], including one where the mean transverse rectal diameter decreased by more than 1 cm after an enema [36]. In the three studies with significant transverse rectal diameter decrease, treatment duration was 1 month [28, 37] or 3 months [26]. However, there was a high heterogeneity in type of laxative treatment, and in treatment and follow-up duration. In addition, not all studies reported treatment response.

## Discussion

This meta-analysis evaluating the diagnostic accuracy of transverse rectal diameter measurement via transabdominal ultrasound shows that transabdominal ultrasound to diagnose functional constipation in children has a sensitivity of 0.68 (95% CI 0.55–0.78) and a specificity of 0.81 (95% CI 0.71–0.88). This is comparable to the diagnostic accuracy for abdominal X-ray for diagnosing constipation [8, 36]. A meta-analysis of studies using transabdominal ultrasound to assess fecal impaction could not be performed due to a limited amount of included studies with high levels of heterogeneity between studies. A significant difference was found in transverse rectal diameter between children with and without functional constipation (1.37 SMD, 95% CI 0.79–1.95; *P* < 0.0001). This indicates that children with functional constipation have a transverse rectal diameter that is on average 1.37 standard deviations wider than that of children without functional constipation. This is considered a large difference [39]. Mean transverse rectal diameter for children with functional constipation was estimated at 3.8 cm ± 0.3 SD, versus 2.2 cm ± 0.2 SD for controls. To account for the different cut-off values used in the included studies, ranging from 2.44 cm to 3.8 cm, the cut-off value for an enlarged rectum was examined as a covariate in the meta-analysis. This covariate is a crucial element in the meta-analysis of diagnostic accuracy, as using a higher cut-off value is expected to correlate with a lower sensitivity and a higher specificity. Surprisingly, a lower or higher transverse rectal diameter cut-off did not have a statistically significant association with the sensitivity or specificity of the model. From a clinical perspective, this seems unlikely. Several factors could have caused this discrepancy. First, only three studies used pre-specified cut-offs for the transverse rectal diameter [15, 34, 35], while all other studies defined the cut-off based on their findings. Studies without pre-defined cut-off tend to overestimate diagnostic accuracy by optimizing the cut-off based on their population. This leads to high heterogeneity in sensitivity and specificity between studies. Second, the majority of included studies had a case–control design. The diagnostic accuracy might be overestimated due to case–control studies using healthy controls and children with alternative diagnoses in whom absence of functional constipation was known prior to ultrasound, reducing the probability of false-positive results [40]. However, one study included control group patients treated with laxatives who no longer met the Rome criteria [34], potentially affecting transverse rectal diameter measurements, as it remains uncertain whether and how long it takes for the transverse rectal diameter to normalize in treated children [41]. It is therefore uncertain if this study might lead to an underestimation of the diagnostic accuracy. Finally, lack of standardized testing methods regarding time to defecation and bladder filling which have been shown to affect transverse rectal diameter potentially skewed the meta-analysis results.

Reported sensitivity and specificity rates for diagnosing fecal impaction via transverse rectal diameter measurement ranged between 68–100% and 83–100%, respectively. The three studies reporting mean transverse rectal diameter in children with and without fecal impaction showed significantly higher mean transverse rectal diameter in children with fecal impaction. Identification of fecal impaction is important before starting treatment because disimpaction improves the response to maintenance therapy [42]. An RCT involving 270 children evaluated the role of transabdominal ultrasound in managing children with functional constipation [43]. The study reported that disimpaction led to considerably better symptom management and compliance in children with transverse rectal diameter larger than 3.0 cm. In children with transverse rectal diameter smaller than 3.0 cm, disimpaction did not result in better symptom management compared to children who did not receive disimpaction [43]. Others however indicated that transverse rectal diameter alone gives an incomplete picture of the severity of constipation and therefore investigated an ultrasound scoring system to assess the extent of fecal loading along the colon in children with constipation [44]. This prospective trial showed that the ultrasound scoring system corresponded well with the self-developed symptom severity score, suggesting a good correlation between symptoms and ultrasound findings. However, both scoring systems were not validated and more research is needed to explore the clinical applicability of this ultrasound scoring system in children with constipation. Nonetheless, the aforementioned studies and the findings of this systematic review suggest that ultrasound holds potential in identifying fecal impaction, and guide treatment strategy in constipated children. Ultrasound’s repeatability makes it useful for monitoring constipation treatment, supporting education, compliance, and dosage adjustments. However, transverse rectal diameter improvements may lag behind symptom relief, requiring further study to assess its role from diagnostic through follow-up.

In the meta-analysis, transverse rectal diameters were significantly larger in children with functional constipation compared to children without functional constipation. Numerous factors could have affected study results and ultrasound measurements. First, an enlarged rectum could correspond to a chronically enlarged rectum due to functional constipation, or to an enlarged rectum due to “normal” filling of the rectum if a child has not defecated for a few hours. A previous study, including 14 healthy children and 14 children under treatment for functional constipation, indeed found that transverse rectal diameter significantly fluctuates pre- and post-defecation (0.7–0.9 cm) in both groups [15]. Transverse rectal diameter can exceed 3 cm in healthy children with imminent defecation urge [15]. Nine studies included in this systematic review reported either the urge to defecate or time to previous defecation, which varied from 2 to 12 h. Due to the heterogeneity of the exclusion criteria and insufficient reporting of time to defecation among included studies, a sensitivity analysis based on time to defecation was not possible, precluding conclusions regarding the effect of time to defecation on rectum size. The variability in transverse rectal diameter related to timing of defecation could be a significant factor accounting for the varying cut-off values and diagnostic accuracy levels observed in the included studies.

Second, many studies neglect to account for age-specific transverse rectal diameter variations. The prevailing standard transverse rectal diameter threshold is 3.0 cm, despite the reasonable expectation that transverse rectal diameter likely varies with age and therefore thresholds should also be age-specific. Unfortunately, little is known about normal transverse rectal diameter sizes in healthy children of different age groups. A prospective cohort study measured transverse rectal diameter with ultrasound of 110 healthy infants at 2 months and 12 months old, reporting means of 1.56 cm and 1.78 cm with upper 95% limits of 2.26 cm and 2.64 cm, respectively [45]. To our knowledge, no other studies investigated standardized values for older healthy children. Three studies reported standardized transverse rectal diameter values including older age groups; however, all used hospital-attending children instead of true healthy controls [24–26]. It is known that defecation parameters, such as stool frequency, colonic transit time, and stool weight, change around the age of 3–4 years in the developmental stages toward achieving fecal continence. This may explain why discrepancies were found in the correlation of age and transverse rectal diameter in this systematic review. The studies finding a positive correlation predominantly included children with a broad age spectrum. The studies describing no correlation included predominantly older children, such as 4 years and older [34, 36] or 6 years and older [28], which may have hindered the ability to show a correlation [24, 25]. Other potential factors include severity and duration of functional constipation complaints, ongoing functional constipation treatments, and frequency of bowel movements. Unfortunately, many studies failed to report relevant variables potentially influencing the evaluation of rectal measurements.

Establishing recommendations for clinical practice based on the findings of this systematic review is challenging. Even though results are promising, to date we cannot recommend transabdominal ultrasound in the diagnosis of functional constipation or fecal impaction, mainly caused by the lack of age-dependent cut-off values and the uncertainty about the impact of the time to defecation, and due to the lack of a standardized protocol in performing ultrasound. One should also consider that the initial diagnosis of functional constipation is mainly based on medical history and physical examination, using the Rome IV criteria. Transabdominal ultrasound might be helpful and complementary in very anxious children in whom the initial diagnosis is uncertain. Another potential clinical application of transverse rectal diameter measurements would be to replace digital rectal examination in identifying a rectal fecal mass in children not meeting two of the Rome criteria and refusing rectal examination. Transabdominal ultrasound could help establish fecal impaction without any radiation exposure.

Future research should focus on the previously mentioned evaluation of transverse rectal diameter measurements in different populations and age groups. Studies should be set up as double-blinded cohort studies. We recommend using a control group similar to the intervention group instead of healthy controls, such as children referred for bowel dysfunction, to prevent overestimation of the diagnostic accuracy. We suggest that future research should separate different age groups in their analyses and use different cut-offs per age group, which need to be determined. With the growing role of point of care ultrasound across various specialties, establishing a standardized protocol for measuring transverse rectal diameter is increasingly important. This standardized protocol should take into account bladder filling, time to defecation, and the urge to defecate. If the urge to defecate is present, it is suggested to postpone the ultrasound to at least 2 h after defecation. Measurements should be performed 2 cm above the symphysis as it is the most commonly used method, and other measurement planes do not seem to have additional value. A full bladder enhances rectum visualization by acting as a lens. Voiding decreases measured transverse rectal diameter and an empty bladder often precludes transverse rectal diameter assessment [15, 29]. Hence, we recommend standardizing ultrasound with a full bladder for consistent results.

The main strength of our study lies in the comprehensive review of the included studies, which involved not only an analysis of outcomes but also an evaluation of factors that could potentially influence their measurements and results. However, our review has several limitations, which primarily stem from the nature of the included studies. The majority of these studies were un-blinded case–control studies that determined the optimal transverse rectal diameter cut-offs based on their own results, likely resulting in an overestimation of the reported diagnostic accuracy. Furthermore, inadequate reporting limited our meta-analysis to a subset of studies. Additionally, many studies failed to report data on inter-observer variability and other relevant variables potentially influencing rectal measurements evaluation. Finally, the exclusion of non-English language studies should be considered.

In conclusion, transabdominal ultrasound holds potential as a non-invasive diagnostic tool for evaluating transverse rectal diameter in assessing functional constipation and identifying fecal impaction in children not meeting at least two of the Rome criteria and refusing rectal examination. However, factors such as age, time to defecation, laxative use, and intra- and inter-observer reliability must be considered when interpreting transabdominal ultrasound. Current literature inadequately addresses these factors, hindering strong recommendations for the role of transabdominal ultrasound in the diagnostic pathway. Future research should focus on establishing age-dependent cut-off values, separating age groups and developing a standardized protocol.

## Supplementary Information

Below is the link to the electronic supplementary material.

Supplementary file1 (DOCX 29.2 KB)

## Data Availability

All our data and meta-data will be made available upon request.
