# Supplementary material for: Accuracy of transabdominal ultrasound to diagnose functional constipation and fecal impaction in children: a systematic review and meta-analysis
Source: Pediatr Radiol. 2024 Nov 15;54(13):2227–42. doi: 10.1007/s00247-024-06083-4 (PMC11638420; doi:10.1007/s00247-024-06083-4)
Supplement: Supplementary file 1 — Supplementary file1 (DOCX 29.2 KB) [file 247_2024_6083_MOESM1_ESM.docx]

**Supplementary Material 1**

**Search strategy**

| Medline search history sorted by search number ascending | | |
| --- | --- | --- |
| # | Searches |  |
| 1 | exp Constipation/ or (Dyschesia or Costiveness or constipat*).ti,ab,kf. |  |
| 2 | rome.ti,ab. |  |
| 3 | 1 or 2 |  |
| 4 | exp ultrasonography, doppler/ or ultrasonography/ or exp ultrasonography, prenatal/ or ultrasonics/ or (ultrasound or ultrasonogra$ or ultrasonic$ or echograph$ or (doppler or duplex) or sonograph$ or sonogram$ or (contrast adj4 US) or echotomograp*).ti,ab,kf. |  |
| 5 | 3 and 4 |  |
| 6 | exp animals/ not humans/ |  |
| 7 | 5 not 6 |  |

| Embase Search history sorted by search number ascending | |  |
| --- | --- | --- |
| # | Searches |  |
| 1 | exp constipation/ or (Dyschesia or Costiveness or constipat*).ti,ab,kw. Or (functional constipation or rome).ti,ab. |  |
| 2 | exp Doppler ultrasonography/ or echography/ or exp fetus echography/ or ultrasound/ or (ultrasound or ultrasonogra$ or ultrasonic$ or echograph$ or (doppler or duplex) or sonograph$ or sonogram$ or (contrast adj4 US) or echotomograp*).ti,ab,kw. |  |
| 3 | 1 and 2 |  |
| 4 | (exp experimental organism/ or animal tissue/ or animal cell/ or exp animal disease/ or exp carnivore disease/ or exp bird/ or exp experimental animal welfare/ or exp animal husbandry/ or animal behavior/ or exp animal cell culture/ |  |
|  | or exp mammalian disease/ or exp mammal/ or exp marine species/ or nonhuman/ or animal.hw.) not human/ |  |

**Supplementary Material 2. QUADAS‐2 tool for assessing methodological quality of included studies**

| **Domain** | **Signaling question** | **Signaling question** | **Signaling question** | **Risk of bias** | **Concerns about applicability** |
| --- | --- | --- | --- | --- | --- |
| **Domain 1: Patient selection** | | | | | |
| Patient selection | Was a consecutive or random sample of patients enrolled? | Was a case-control design avoided? | Did the study avoid inappropriate exclusions? | Could the selection of patients have introduced bias? | Is there concern that the included patients do not match the review question? |
|  | Yes: if all consecutive or random samples of patients were enrolled  No: if convenience or selected samples were enrolled  Unclear: if this was not clear from the report | Yes: if data was collected from a prospective sample  No: if a case-control study was conducted  Unclear: if this was not clear from the report | Yes: if the study avoided inappropriate exclusions  No: if patients were excluded inappropriately (e.g. history of constipation, individuals who had undergone previous treatment, ethnicity)  Unclear: if this was not clear from the report | Low: if "Yes" for all signaling questions  High: if "No" was reported for at least 1 signaling question  Unclear: if "Unclear" was reported for at least 1 signaling question | Low: if the included population consists of patients irrespective of age and sex, and if inappropriate exclusions were avoided  High: if study authors used inappropriate exclusions  Unclear: if insufficient information was available to make a judgement |
| **Domain 2: Index tests** | | | | | |
| Index test ‐ ultrasound to measure rectal diameter | Were index test results interpreted without knowledge of the results of FC diagnosis? | If threshold was used, was it prespecified? |  | Could the conduct or interpretation of the index test have introduced bias? | Is there concern that the index test, its conduct, or interpretation differ from the review question? |
|  | Yes: if rectal diameter was measured without knowledge of symptoms or diagnosis  No: if rectal diameter was measured with knowledge of symptoms or diagnosis  Unclear: if this was not clear from the report | Yes: if criteria for positive findings on ultrasound were prespecified  No: if criteria for positive findings on ultrasound were not prespecified  Unclear: if this was not clear from the report |  | Low: if "Yes" for all signaling questions  High: if "No" was reported for at least 1 signaling question  Unclear: if "Unclear" was reported for at least 1 signaling question | Low: if ultrasound was performed without knowledge of FC symptoms or diagnosis  High: if ultrasound was performed after knowledge of FC symptoms or diagnosis  Unclear: if insufficient information was available to make a judgement |
| **Domain 3: Reference standard** | | | | | |
| Reference standard ‐ diagnosis of FC | Is the reference standard likely to correctly classify the target condition? | Were the reference standard results interpreted without knowledge of the results of index test? |  | Could the reference standard, its conduct, or its interpretation have introduced bias? | Is there concern that the target condition as defined by the reference standard does not match the review question? |
|  | Yes: if an acceptable reference standard, such as Rome IV criteria, was used  No: if patients did not undergo an acceptable reference standard  Unclear: if this was not clear from the report | Yes: If symptoms and FC diagnosis were interpreted without knowledge of the results of ultrasound  No: If symptoms and FC diagnosis were interpreted with knowledge of results of ultrasound  Unclear: If this was not clear from the report |  | Low: if "Yes" for all signaling questions  High: if "No" was reported for at least 1 signaling question.  Unclear: if "Unclear" was reported for at least 1 signaling question | Low: If an acceptable reference standard was used, and if diagnosis was made without knowledge of symptoms  High: If an acceptable reference standard was not used, or if diagnosis was made after ultrasound measurement  Unclear: If insufficient information was available to make a judgement |
| **Domain 4: Flow and timing** | | | | | |
| Flow and timing | Was there an appropriate interval between index test and reference standard? | Did all patients receive a reference standard? | Were all patients included in the analysis? | Could patient flow have introduced bias? |  |
|  | Yes: if diagnosis and ultrasound were performed sequentially within a week  No: if diagnosis and ultrasound were not performed within 1 week  Unclear: if this was not clear from the report | Yes: if all patients were assessed to determine whether they fulfilled criteria for FC  No: if some patients’ symptoms were not assessed  Unclear: if this was not clear from the report | Yes: if all patients were included in the final analysis  No: if not all patients were included in the final analysis  Unclear: if this was not clear from the report | Low: if "Yes" for all signaling questions  High: if "No" was reported for at least 1 signaling question  Unclear: if "Unclear" was reported for at least 1 signaling question |  |

*FC* functional constipation
